# Regulatory mechanism and research progress of ferroptosis in obstetrical and gynecological diseases

**DOI:** 10.3389/fcell.2023.1146971

**Published:** 2023-03-30

**Authors:** Xinyue Wang, Yanchen Wei, Fangyi Wei, Haibin Kuang

**Affiliations:** ^1^ Department of Physiology, Basic Medical College, Nanchang University, Nanchang, China; ^2^ Department of Clinical Medicine, School of Queen Mary, Nanchang University, Nanchang, China; ^3^ Jiangxi Provincial Key Laboratory of Reproductive Physiology and Pathology, Nanchang University, Nanchang, China

**Keywords:** ferroptosis, preeclampsia, polycystic ovarian syndrome, regulatory mechanism, endometriosis (EMs)

## Abstract

Ferroptosis is a novel type of regulated cell death driven by iron-dependent lipid peroxidation, which is distinguished from traditional types of programmed cell death, such as apoptosis, proptosis and necrosis et al. Impaired iron homeostasis, lipid peroxidation and antioxidants depletion are three hallmarks of ferroptosis. Over the past years, emerging studies support the notion that ferroptosis might be involved in the pathology of obstetrical and gynecological diseases, including preeclampsia (PE), endometriosis (EMs) and polycystic ovarian syndrome (PCOS). In the PE condition, the high sensitivity of trophoblasts towards ferroptosis has been found to potentially link to inflammation, suboptimal vascular remodeling and aberrant hemodynamics, which are three prominent pathophysiological features of PE. As for EMs, compromised ferroptosis of endometrial cells was associated with the formation ectopic lesions, whereas in the nearby lesions, the presence of ferroptosis was suggested to promote the progression of EMs, contributing to the relative clinical manifestations. Ferroptosis has been implicated a crucial role in the initiation of ovarian follicular atresia, which might help to manage ovulation in PCOS patients. Taken together, this review explored the basis of ferroptosis mechanisms and comprehensively summarized the latest discovery of roles of ferroptosis on PE, EMs and PCOS, gaining a deeper insight into the pathogenesis of these obstetrical and gynecological diseases and investigation of novel therapeutic interventions.

## 1 Introduction

Obstetrical and gynecological diseases are a large group of diseases which can be classified into different categories based on their anatomical structures, such as placenta, endometrium and ovary. These diseases are the main threat to female physical wellbeing, the occurrence of which is closely related to the death of reproductive cells. Understanding the molecular mechanism of cell death is of great importance as it might facilitate better defining obstetrical and gynecological disease pathogenesis and further shed light on relative therapeutic strategies.

Ferroptosis, first proposed by Dixon et al., in 2012, is a novel type of regulated cell death that is driven by iron-dependent lipid peroxidation (Dixon et al., 2012). It is distinguished from conventional programmed cell death pathways, such as apoptosis, autophagy and necrosis (Dixon et al., 2012). Ferroptosis is characterized by the intersection of iron overload, elevated lipid peroxidation and antioxidants depletion, presenting with membrane-containing subcellar organelle abnormalities, such as mitochondrial shrinkage and plasma membrane rupture (Yang and Stockwell, 2008; Dixon et al., 2012; Stockwell, 2022).

Currently, emergent evidence suggests that ferroptosis might be implicated in the progression of certain obstetrical and gynecological diseases. As the presence of events females might encounter in life, including menstruation, pregnancy and lactation, iron homeostasis in women is more delicate than men (Eschbach, 2005). Additionally, the normal function of the female reproductive system is significantly influenced by oxidative stress (OS), which is kept in a balance between reactive oxygen species (ROS) and antioxidants, whereas tipping the balance to OS side either due to overproduction of ROS or antioxidants depletion results in numerous obstetrical and gynecological conditions (de Bruin et al., 2002; Fainaru et al., 2002; Mocatta et al., 2004).

Among these obstetrical and gynecological conditions, settings associated with ferroptosis were found to be closely linked to preeclampsia (PE), endometriosis (EMs) and polycystic ovary syndrome (PCOS). Recently, more evidence has suggested the pathogenesis of PE is related to ferroptosis of trophoblasts, which might cause inflammatory responses, hypoxia-reperfusion injury, shallow invasion of trophoblasts and suboptimal spiral artery remodeling (Orlov et al., 2016; Ng et al., 2019; Chen et al., 2022). It is considered that ferroptosis is inhibited at the ectopic lesion in EMs, which allows the endometrial cells to survive and grow in the iron overload and excessive OS condition (Ng et al., 2020). In contrast, these conditions trigger ferroptosis of the normal nearby tissues which causes adverse clinical outcomes and clinical manifestations (Chen et al., 2021a; Li et al., 2021a; Li et al., 2022; Ni et al., 2022).

Ferroptosis was suggested to be involved in the initiation of normal follicular atresia, which is compromised under the PCOS condition, thereby induction of ferroptosis might provide a novel therapeutic strategy for this disease (Rabahi et al., 1999; Terenina et al., 2017; Zhang et al., 2018; Cui et al., 2022).

Given this, we summarize the current knowledge related to the regulatory mechanism of ferroptosis and its associated pathways, including iron metabolism, antioxidants depletion and lipid metabolism. Following that, we further explore and discuss the roles of ferroptosis in PE, EMs and PCOS, which might provide reference to further investigation of therapeutic interventions for these obstetrical and gynecological diseases.

## 2 Molecular mechanisms of ferroptosis

**FIGURE 1:**
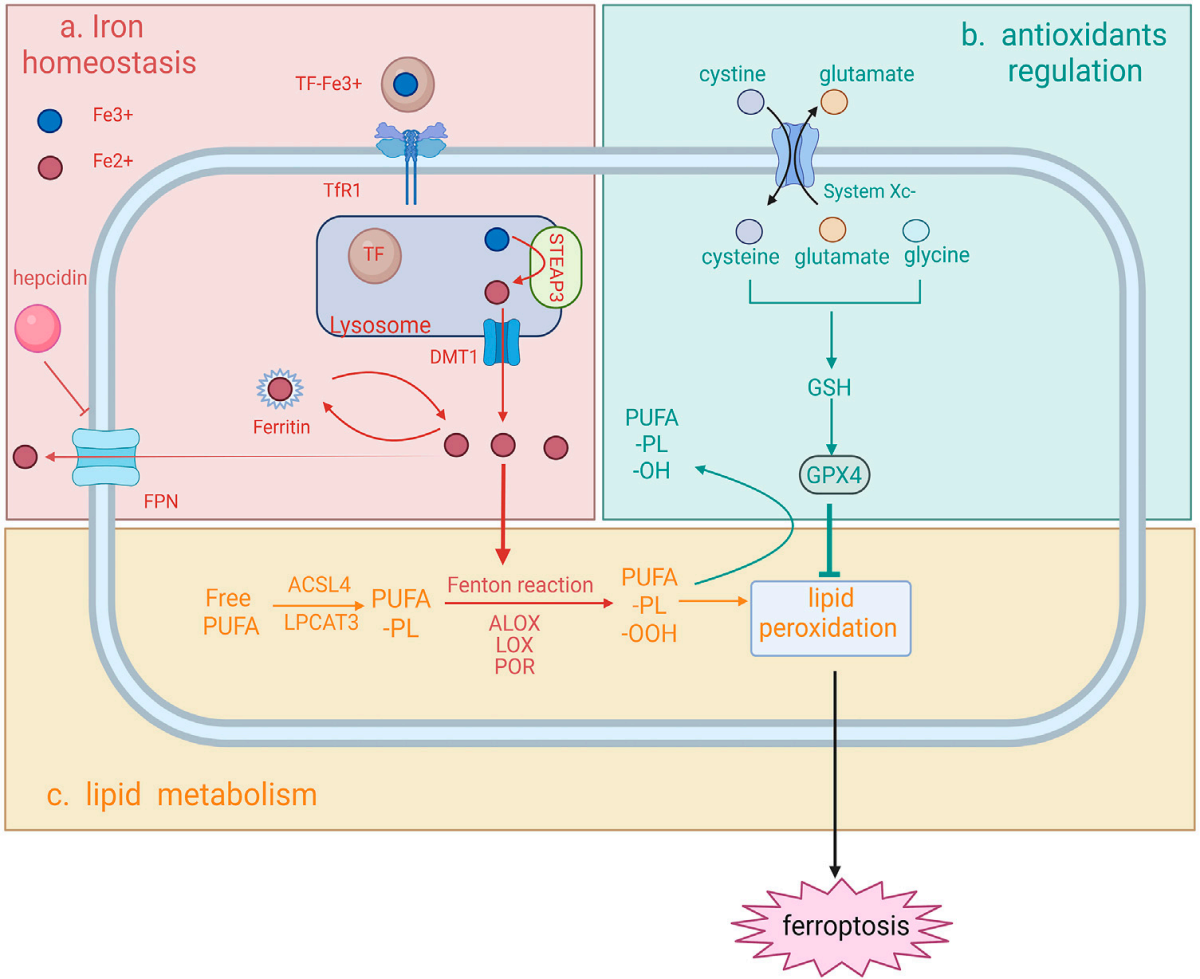
Schematic representation of the molecular mechanism of ferroptosis ferroptosis can be characterized by the intersection of iron homeostasis **(A)**, antioxidants regulation **(B)** and lipid metabolism **(C)**. **(A)** Iron homeostasis (top left). Iron is first internalized in the form of TF-Fe^3+^ *via* TfR1 and this complex dissociates once entering the lysosome. Fe^3+^ is reduced into Fe^2+^ by STEAP3 and further transported outside the lysosome. In the cytosol, Fe^2+^ is either stored in the form of DMT1 or exported by FPN. FPN can be inhibited by hepcidin, thus regulating cytosolic iron availability. In the process of ferroptosis, the impaired iron import, storage or export contributes to the iron overload and consequently promotes iron-dependent Fenton reaction and the action of iron-dependent enzymes and thereby lipid peroxidation. **(B)** Antioxidants depletion (top right). GPX4, a key antioxidant in ferroptosis, is responsible for the reduction of toxic lipids and its depletion leads to the failure of the suppression of lipid peroxidation. **(C)** Lipid metabolism (at the bottom). For lipid peroxidation to occur, free PUFA needs to be incorporated into phospholipid to form membrane-bounded PUFA-PL. The following Fenton reaction mediated by an excessive iron together with insufficient antioxidant activity contributes to the overproduction of PUFA-PL-OOH and finally ferroptotic cell death (created with Biorender.com).

Ferroptosis was characterized as a novel form of iron-dependent, non-apoptotic cell death. Following a decade of continuous investigation, the understanding and knowledge of the molecular mechanism of ferroptosis have been largely broadened to an intersection of iron homeostasis, antioxidants regulation and lipid metabolism (Stockwell, 2022) (Figure 1).

### 2.1 Impaired iron homeostasis

Iron is an indispensable metal regulating the sensitivity to ferroptosis, since iron is required for the membrane-bound lipid peroxidation *via* the Fenton reaction or the action of iron-dependent enzymes (Yang et al., 2016; Zou et al., 2020). Therefore, impaired iron homeostasis might trigger ferroptosis and cause several diseases. Iron is transported in the circulation *via* iron transporter transferrin (TF) in the form of TF-Fe^3+^ (Wallace, 2016). TF-Fe^3+^ is internalized into the target cell *via* endocytosis after binding to the transferrin receptor 1 (TFR1). Fe^3+^ is then released from TF, reduced to Fe^2+^ *via* the ferrireductase activity of six-transmembrane epithelial antigen of prostate 3 (STEAP3), and enters the cytosol by divalent metal transporter 1 (DMT1). In non-erythroid cells, most of the cytosolic iron is stored in ferritin (Arosio and Levi, 2010), and excess iron is exported by ferroportin (FPN) (Donovan et al., 2005). The abundance of iron is regulated by hepcidin, the molecule that can bind to FPN with the presence of high iron concentration, thereby preventing iron release into the bloodstream (Ganz and Nemeth, 2012).

Impaired iron homeostasis *via* dysregulating any one of iron import, storage or export process can alter the sensitivity of ferroptosis. Suppressing the expression of *TFRC*, the gene encoding TFR1, prevents cells from ferroptosis after treating with ferroptosis inducer erastin (Gao et al., 2015). Degradation of ferritin by nuclear receptor coactivator 4 (NCOA4)-mediated ferritinophagy has been demonstrated to cause iron overload and induce ferroptosis (Hou et al., 2016). In contrast, overexpression of ferritin by suppressing the expression of iron response element binding protein 2 (IREB2) has been found to inhibit erastin-induced ferroptosis (Gammella et al., 2015). Additionally, studies reported that knockdown of FPN led to iron overload and accelerated erastin-induced ferroptosis in neuroblastoma cells (Geng et al., 2018).

Iron overload causes an escalating membrane-bound lipid peroxidation *via* the Fenton reaction, increasing the susceptibility to ferroptosis. It is noteworthy that iron-dependent enzymes, such as arachidonate lipoxygenases (ALOXs), lipoxygenase (LOX) and cytochrome P450 oxidoreductase (POR), can also promote lipid peroxidation and further ferroptosis, by initiating the formation of lipid peroxides which are the substrate of the Fenton reaction (Zou et al., 2020).

### 2.2 Antioxidants depletion

Antioxidants are the defenders counteracting the phospholipid hydroperoxides produced from excessive iron in cells, thus protecting against ferroptosis. As a strong antioxidant and ferroptosis inhibitor, glutathione (GSH) plays a critical role in hindering cells from the damage of ROS. Ferroptosis is prevented only with sufficient GSH storage. Under physiological status, intracellular cysteine (reduced from cystine), glutamate and glycine are three building blocks for GSH construction. Sodium-independent, anion amino acid transport system (System Xc^−^) is a heterodimer consisting of two subunits, SLC7A11 and SLC3A2. System Xc^−^ serves as an antiporter to import cystine into the cytoplasm, promoting the GSH synthesis and further the production of glutathione peroxidase 4 (GPX4). GPX4, a selenium-centered ferroptosis inhibitor, can convert toxic polyunsaturated fatty acid phospholipid hydroperoxides (PUFA-PL-OOH) into non-toxic PUFA phospholipid alcohols (PUFA-PL-OH), thereby combating the oxidation of the membrane-localized lipid peroxidation. Depletion of GPX4 and system Xc^−^ leads to a continuous accumulation of intracellular lipid peroxides and further ferroptosis. Glutamate is also an important factor influencing ferroptosis as it is also transported by system Xc^−^, the concentration of which affects the function of the antiporter. Increased extracellular glutamate diminishes the exchange rate, thus leading to a shortage of intracellular cystine and thereby ferroptosis.

### 2.3 Lipid metabolism

As the driving force of ferroptosis, both impaired iron homeostasis and depletion of antioxidants eventually lead to the accumulation of lipid hydroperoxides into an overwhelming level. For lipid peroxidation to occur, PUFA, a key substrate for peroxidation is needed to be incorporated into specific membrane phospholipids (PLs), such as arachidonic acid (AA) and adrenic acid (Yang et al., 2016), to form membrane phospholipids (PUFA-PLs). In this process, acyl-CoA synthetase long chain family member 4 (ACSL4) and lysophosphatidylcholine acyltransferase 3 (LPCAT3), are responsible for the successful localization of PUFA to the cell membrane by esterification of PUFA to PUFA-PL, thus enabling the lipid peroxides to execute its cytotoxicity function (Ursini et al., 1982). Following the successful attachment of PUFA to the plasma membrane, PUFA-PL will be oxidized to toxic PUFA phospholipid hydroperoxides (PUFA-PL-OOH) *via* iron-dependent peroxidase and iron-mediated Fenton reaction as aforementioned. Moreover, the insufficient oxidative defense mediated by the depletion of ferroptosis inhibitors has also been implicated in the occurrence of ferroptotic cell death. The fatal accumulation of lipid peroxidates underlies several morphological alterations, including membrane structure modifications and increased membrane permeability, which ultimately contribute to plasm membrane rupture (Yu et al., 2021). Lipid peroxidates can be further decomposed into toxic derivatives, such as serum malondialdehyde (MDA) and 4-hydroxynonenal (4-HNE). These two by-products were reported as the major toxic by-products, which could trigger the crosslinking formation of protein-DNA bases and influence protein function, contributing to cell damage and consequently ferroptotic cell death (Gaschler and Stockwell, 2017; Feng and Stockwell, 2018).

## 3 The roles of ferroptosis in PE

PE is a placenta-associated disease characterized by hypertension with the damage of maternal end organs during the period of gestation with a prevalence of 5%–7% among pregnant women (Rayman et al., 2002). It is one of the major contributing factors to both maternal and fetal mortality and morbidity (Witcher, 2018). Although the exact pathophysiological mechanism of this severe pregnancy-associated disease remains elusive, recent studies have suggested that ferroptosis is potentially involved in the pathology of PE, especially the early-onset type of PE.

### 3.1 Alteration of ferroptosis regulators under the PE condition

Intriguingly, numerous studies have found that molecular alterations of placenta cells under PE conditions are closely related to the molecular mechanism of ferroptosis, including abnormal lipid peroxidation, depletion of intracellular antioxidants and impaired iron homeostasis. These three aspects will be explored as follows.

#### 3.1.1 Abnormal lipid peroxidation

During embryo implantation and early placentation of healthy pregnancy, increased ROS can be seen in the physiological process including hypoxia-reoxygenation and physiological stress response due to the maintenance of fetal development (Burton et al., 2021). In healthy gestation, ROS generation is successfully controlled by adequate antioxidants, thus hindering trophoblasts from the damage of OS (Schoots et al., 2018). Whereas in PE, given the presence of compromised fetal placenta circulation, the insufficient placenta perfusion gives rise to restrained oxygen supply to the cellular electron transport chain, consequently soaring the production of ROS and toxic lipid peroxides into an overwhelming level (Aouache et al., 2018).

Numerous studies have elucidated the involvement of abnormal lipid peroxidation in PE conditions. Enriched PUFA, especially AA, has been reported in the trophoblasts of PE patients (Brown et al., 2016). Additionally, metabolomic analysis has revealed the presence of a significantly higher mitochondrial level of PUFA among PE patients, further validating the accumulation of PUFA (Chen et al., 2022). Moreover, the overexpression of several genes involved in disrupted lipid metabolism has been identified, including LPCAT3, ACSL4, which are two key gene products for membrane localization of PUFA to form PUFA-PL (Dixon et al., 2015; Ou et al., 2016; Kagan et al., 2017). Furthermore, single-cell sequencing of placental trophoblasts, endothelial cells and mesenchymal fibroblasts showed the overexpression of LOX-5, LOX-15 and spermidine/spermine N1-acetyltransferase 1 (Sat1), which are key enzymes to trigger membrane lipid peroxidation (Dixon et al., 2015; Ou et al., 2016; Kagan et al., 2017). As such, MDA was also reported to be notably upregulated in gestational women who undergo PE in several investigations (Siddiqui et al., 2013; Yang et al., 2020; Chen et al., 2022). Collectively, the level of membrane-bound lipid peroxidation is significantly upregulated and OS level is escalated in trophoblasts under PE conditions.

#### 3.1.2 Depletion of intracellular antioxidants

The downregulated level of GPX4 has been found in PE patients compared with women with normal pregnancy (Mistry et al., 2010; Roland-Zejly et al., 2011). The allelic gene analysis conducted by Peng et al. further reported that rs713041 in GPX4 is more associated with PE, especially the early-onset type of PE (Peng et al., 2016). In addition, the level of selenium has been found to be negatively associated with the risk of PE. Of note, supplementation of selenium decreases the PE incidence during pregnancy, suggesting a context dependence on the requirement of selenium-containing GPX4 on PE pathology (Proneth and Conrad, 2019). PLA2G6, an enzyme that removes oxidized PUFA from membrane lipids, has been recently proposed for its role in ferroptosis defense (Balboa and Balsinde, 2006; Beharier et al., 2020; Chen et al., 2021b). In the rat model, the deletion of PLA2G6 expression in trophoblastic cells together with GPX4 inhibition results in embryo lethality in most cases of pregnant murine. Notably, the action of PLA2G6 is exerted only following the exhausted GPX4, suggesting PLA2G6 serves as the second line molecule in ferroptosis defense (Beharier et al., 2020). Taken together, the depletion of intracellular antioxidants protecting against ferroptosis is present in PE conditions.

#### 3.1.3 Impaired iron homeostasis

It has been estimated that approximately 18% of pregnant women with PE present with dramatically elevated TF-saturation, indicating the presence of iron overload in PE patients (Liu et al., 2019). A meta-analysis conducted by Chen et al. revealed that the hepcidin expression increased during the third trimester in women with PE, which is opposite to the normal pregnancy (Chen et al., 2021b). There is a possibility that hepcidin overexpression serves as the protective mechanism to counter the iron-dependent cytotoxicity, which might be contributed by either excessive iron supplementation or the oxidative damage-mediated red cell rupture (Liu et al., 2016). In addition, patients with PE were reported to present an overexpressed level of maternal TF and ferritin, and downregulated expression TFR (Rayman et al., 2002; Liu et al., 2019). The excessive iron further favors lipid peroxidation *via* the Fenton reaction and increases the vulnerability of ferroptosis.

To sum up, conditions of upregulated membrane-bound lipid peroxidation, reduced antioxidants and excessive iron accumulation are present in trophoblasts under the PE condition, suggesting increased susceptibility of trophoblasts to ferroptosis and a plausible correlation between PE and ferroptosis.

### 3.2 The roles of ferroptosis in suboptimal spiral artery remodeling in PE

#### 3.2.1 Ferroptosis suppresses trophoblast differentiation

In the process of placentation, differentiation of cytotrophoblast cells into either syncytiotrophoblast cells or extravillous trophoblast cells (EVTs) is crucial for proper spiral artery remodeling. This ultimately contributes to hyperoxia and support fetal growth (Nicolas et al., 2002; Bauer and Lip, 2017; Bah et al., 2017). Accumulative studies have suggested that ferroptosis might affect the internal regulation of trophoblast differentiation under pathological conditions. It is demonstrated that the exposure of RSL3, a ferroptosis inducer, is associated with the diminished production of chorionic gonadotropin and placental lactogen in the primary human trophoblast (PHT) cell line, which are two hormones required for the differentiation of the cytotrophoblasts into syncytiotrophoblasts. This suggests the induction of ferroptosis can suppress trophoblast differentiation (Sangkhae et al., 2020). cAMP and PKA are two crucial molecules involved in trophoblast differentiation. It has been observed that RSL3 induction leads to the accumulation of PUFA-PL and insufficiency of cAMP and PKA in trophoblasts, subsequently resulting in oxidative damage and placenta function impairment (Bauer and Lip, 2017; Liu et al., 2019). Moreover, ferroptosis was triggered in the presence of lower dose exposure of RSL3 in trophoblast cells, suggesting a diminished ferroptosis activating threshold might be present in the trophoblast cell with higher vulnerability (Beharier et al., 2020).

#### 3.2.2 Suboptimal remodeling of spiral arteries

During the period of a healthy pregnancy, EVTs firstly migrate to maternal decidua and form endovascular plugs to prevent oxygen supply into the intervillous space during the period of 8–10 weeks of gestation (Liu et al., 2018). This consequently leads to hypoxia of fetal tissue, favoring cytotrophoblast cells undergoing proliferation to enlarge the pool for further differentiation (Genbacev et al., 1996; Genbacev et al., 1997). Following 2 weeks, differentiated EVTs remodel maternal spiral arteries by destructing and replacing maternal vascular endothelial cells, contributing to the loss of smooth muscle cells and their innervated autonomic nerves. This in turn leads to diminished vessel wall reactivity, elevated vasodilation and declined vessel resistance, and ultimately hyperoxia (Burton et al., 2009). Consequently, the oxygen provision significantly increases to support fetal growth.

**FIGURE 2:**
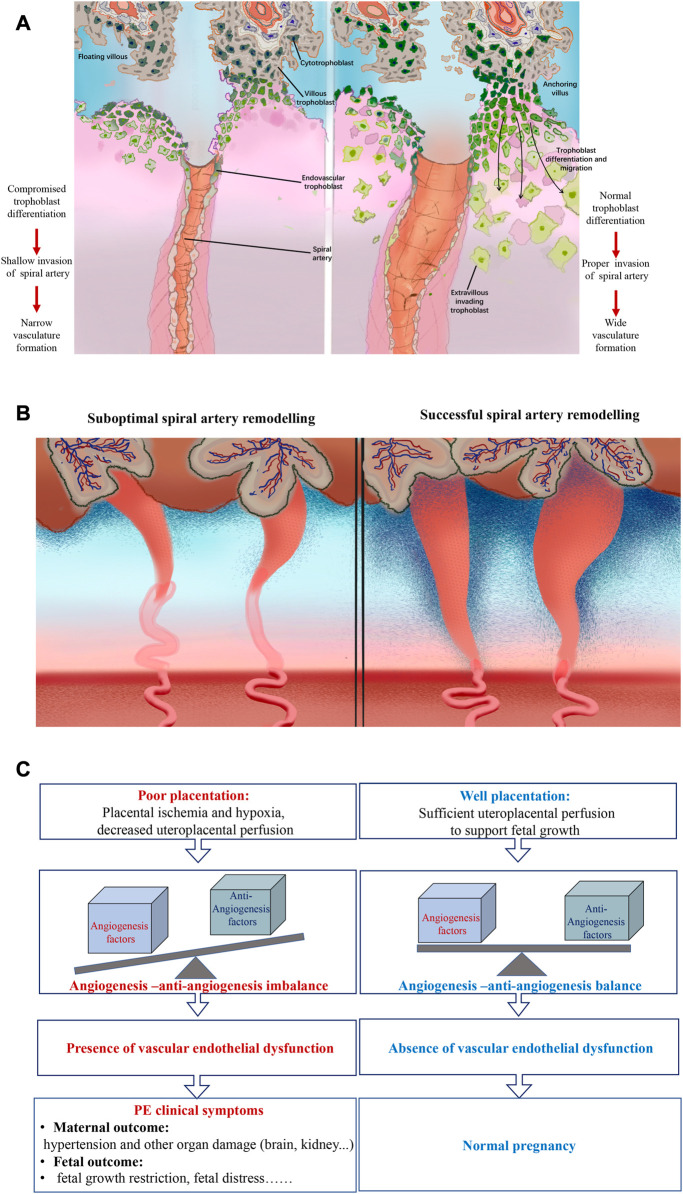
Schematic overview of the pathology of PE (left) and normal physiology of pregnancy (right) **(A)** Diagram of trophoblast invasion at the fetal-maternal interface. Under the PE condition, ferroptosis potentially mediated compromised cytotrophoblast cell differentiation leads to the shallow invasion of spiral artery remodeling and further narrow vasculature formation. Whereas in normal pregnancy, the physiological and normal differentiation of trophoblast contributes to the proper spiral artery construction and wide vasculature architecture. **(B)** Schematic representation of the model of spiral artery remodeling of PE condition and normal pregnancy. Compared with normal and physiological pregnancy, the spiral artery remodeling is less sufficient and adequate under PE conditions as the result of narrow and thick-walled spiral arteries formation. **(C)** Diagram of the suboptimal remodeling resulted PE pathophysiology and physiology of normal pregnancy. Compared with a healthy pregnancy, PE was characterized by placenta ischemia and hypoxia and decreased uteroplacental perfusion, known as poor placentation. This further leads to angiogenesis and anti-angiogenesis imbalance and consequently vascular endothelial dysfunction. Finally, the characterized PE clinical symptoms will present both in pregnant women manifested as multi-system affected damage and in neonates presented as adverse birth outcomes.

Suboptimal remodeling of spiral arteries is found under PE conditions. As aforementioned, the differentiation of trophoblasts is inhibited by ferroptosis, which might be the reason for impaired remodeling. Furthermore, additional risk factors including prior PE, chronic hypertension, and gene susceptibility, contribute to hyper-peroxidation on trophoblast cell membranes, further suppressing the differentiation of trophoblast cells in the maternal-fetal interface (Bartsch et al., 2016; Vennou et al., 2020). This leads to a compromised invasion of trophoblasts to maternal decidual and forming narrow, thickened maternal spiral arteries instead (Ng et al., 2019). Additionally, the resultant placental ischemia and under-perfusion further contribute to the release of angiogenic factors into the maternal circulation (Ngene and Moodley, 2018). Apart from the involvement of ferroptosis-mediated hypoxia in PE, the alteration of hemodynamic pattern has been proposed in PE pathology (Burton et al., 2009). Three-dimensional reconstructions suggest that in PE, due to the shallow invasion of the spiral artery, flow with fast and turbulent hemodynamical properties is present (Young et al., 2012; Bauer and Lip, 2017; Sangkhae et al., 2020). These factors significantly change maternal systemic endothelial function and further lead to several systemic syndromes and adverse pregnancy outcomes (Figure 2).

### 3.3 The role of ferroptosis cross-linked inflammation in PE pathology

PE is defined as the excessive inflammatory response along with the overexpression of pro-inflammatory cytokines and damage-associated molecular patterns (DAMPs), presenting with multi-system-affected clinical manifestations, such as hypertension, headache, proteinuria, visual disturbance, and upper abdominal discomfort (Shah et al., 2008; Macdonald-Wallis et al., 2011; Errera et al., 2013; Jentho and Weis, 2021). Recently, given the great advances made in the understanding of the PE molecular mechanism, an increasing number of evidence has pointed out that ferroptosis might couple with inflammation.

#### 3.3.1 Expression of pro-inflammatory factors

Studies have demonstrated that cells undergoing ferroptosis release pro-inflammatory DAMPs to induce innate immunity (Jentho and Weis, 2021). Additionally, overexpression of ferroptosis inhibitors is associated with the reduced activation level of inflammasome NLRP1 and NLRP3, which activates the downstream cascade and ultimately diminishes the production of interleukin-1β (IL-1β), whereas the opposite effects were shown with applying ferroptosis activators (Miao et al., 2010). Moreover, the inflammation level is positively associated with ferroptosis, supported by the evidence that silencing the NLRP1 inflammasome contributes to the decreased level of ferroptosis (Meihe et al., 2021).

#### 3.3.2 Inhibition of GPX4 irritates inflammation

GPX4 is known to reduce the pro-inflammatory products generated by LOX and prostaglandin-endoperoxide synthase (PTGS), which in turn counters inflammatory response (Huang et al., 1999). In PE patients, the diminished GPX4 level in the placenta mitigates the reduction of these pro-inflammatory mediators, thus activating innate immunity and promoting inflammation towards the placenta. Therefore, the synergetic effects of ferroptosis and inflammatory response might contribute to PE pathology (Proneth and Conrad, 2019).

### 3.4 Ferroptosis, aberrant hemodynamics and inflammation-the vicious cycle in PE pathology

**FIGURE 3:**
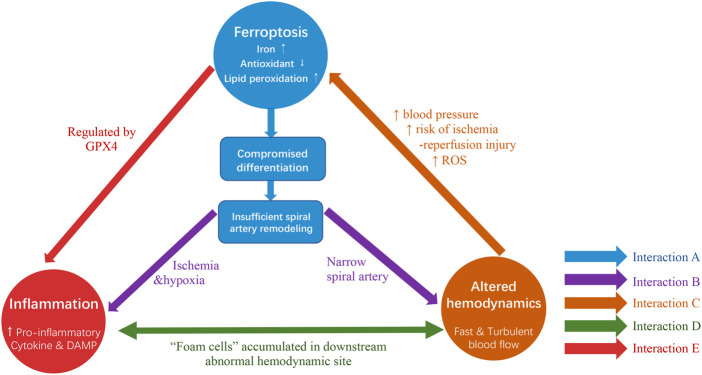
The interactions among ferroptosis, inflammation and altered hemodynamics co-contribute to the pathogenesis of PE. (A) Ferroptosis, characterized by iron overload, antioxidants depletion and lipid peroxidation, is potential to contribute to compromised trophoblast cell differentiation and consequently results in suboptimal spiral artery remodeling. (Arrows shown in blue). (B) On one hand, ferroptosis resultant suboptimal remodeling exposes ischemia and hypoxia environment to the placenta and poses threats to the inflammatory response. On the other hand, inadequate spiral artery remodeling characterized by narrow spiral artery formation leads to altered hemodynamics (Arrows shown in purple). (C) This fast and turbulent blood flow is likely to promote ferroptosis, as it might contribute to high maternal blood pressure, increased risk of ischemia-reperfusion injury and soared ROS level (Arrow shown in orange). (D) Inflammatory response and altered hemodynamics are possible to be inter-connected as evidence suggested the existence of “foam cells” accumulated in downstream abnormal hemodynamic locations (Arrow shown in green). (E) An intimate relationship might be exhibited between inflammation and ferroptosis due to the presence of GPX4. (Arrows shown in red).

Based on the previous study with an in-depth analysis of the roles of ferroptosis in PE, a vicious cycle among ferroptosis, inflammation and aberrant hemodynamics is proposed in the pathology of PE (Figure 3). Firstly, emergent evidence suggested ferroptosis might involve in the pathogenesis of suboptimal spiral artery remodeling which leads to aberrant hemodynamics of maternal-fetal circulation with fast and turbulent blood flow (Young et al., 2012; Bauer and Lip, 2017; Sangkhae et al., 2020). Such hemodynamics subsequently poses the risk on ischemia-reperfusion injury and escalates OS, thus turning to the increased susceptibility of ferroptosis to trophoblasts (Hung et al., 2001). Secondly, the hypoperfusion and hypoxia caused by suboptimal spiral artery remodeling contribute to placental dysfunction and the production of inflammatory factors in the placenta (Redman et al., 1999). Besides, inflammation could also be directly triggered by ferroptosis *via* the release of pro-inflammatory DAMPs or inhibition of GPX4 (Proneth and Conrad, 2019; Jentho and Weis, 2021). Thirdly, the histological examination of spiral artery walls in PE patients found an accumulation of macrophages-derived “foam cells” in its interior wall located downstream of defective remodeled spiral arteries, similar to the morphological features of the early stage of atherosclerosis. This indicates that the fast and turbulent flow is possible to induce vascular lesions with the formation of foam cells and inflammatory response (Staff et al., 2022). However, the mechanism underlying the formation of foam cells is still unclear, several studies suggest that this might be involved in ferroptosis-associated lipid peroxidation in trophoblasts due to the presence of lipid-enriched foam cells (Alahari et al., 2018; Chen et al., 2022). Taken together, the intimate relationship is present in ferroptosis, inflammation and altered hemodynamics. The mutual interactions among these three elements enable them to work synergistically to aggravate placenta injuries and finally contribute to the pathology of PE.

## 4 The roles of ferroptosis in EMs

EMs is a chronic inflammatory disorder characterized by the abnormal growth of endometrial tissue outside of the uterus, causing severe pelvic pain and infertility (As-Sanie et al., 2019). Although the exact etiology remains elusive, several factors associated with the pathogenesis of EMs have been identified, including retrograde menstruation, OS, inflammation and hormones. (Matarese et al., 2003; Zondervan et al., 2018; Zondervan et al., 2020). Studies suggested that ferroptosis acted as a double-edged sword that not only provides a novel treatment strategy, but also involved in the EMs progression.

### 4.1 Formation of ectopic endometrial lesions

Excessive iron, ROS, and free radicals were reported in the microenvironment of ectopic endometrial lesions, contributed by inflammation, repetitive bleeding, and gradual accumulation of menstrual debris (Nisolle and Donnez, 1997; Murphy et al., 1998; Scutiero et al., 2017). However, endometrial cells from retrograde menstrua can still survive and proliferate under the over-exposure to a ferroptosis-favoring environment, suggesting ferroptosis might be compromised in the ectopic endometrial stromal cells (EESCs) (Ng et al., 2020). Li et al. demonstrated that the expression of ferroptosis-associated genes was altered in the ectopic endometrium, which hinders the ferroptosis pathway (Li et al., 2021b). NCOA4, an identified receptor mediating ferritinophagy, was found to be diminished in the EESCs, consequently reducing intracellular iron content (Goodall and Thorburn, 2014). It has been demonstrated that the upregulated lncRNA ADAMTS9 expression in EESCs contributes to the overexpression of GPX4 by sponging miR-6516-5p, thereby promoting the antioxidation of endometrial lesions and preventing ferroptosis (Wan et al., 2022). In addition, the downregulation of Sat1 and ALOX was suggested to reduce the membrane lipid peroxidation in the ectopic endometrial tissue (Ou et al., 2016). Moreover, a study has observed that the mitochondria located at the inner cyst wall of ectopic endometrial lesions present shrunken size, increased membrane density and reduced mitochondrial cristae, similar to the appearance of ferroptosis-affected mitochondria, whereas no significant alterations have been found in the outer layer of ectopic EMs. This distinguished pattern indicates that the different resistance to ferroptosis might exist in the ectopic endometrial wall and further contribute to ectopic lesions (Li et al., 2022). Collectively, the alterations aforementioned reduce the vulnerability of EESCs to ferroptosis and enable EESCs to survive and form ectopic lesions under the condition of excessive OS and iron overload.

Intriguingly, it has been reported that EESCs are more susceptible to ferroptosis induction therapy compared with normal endometrial stromal cells, implying the promising future of the reactivation of ferroptosis for patients with EMs (Li et al., 2021c).

### 4.2 Progression of endometrial lesions contributed by ferroptosis

Ferroptosis coupled with the inflammatory and angiogenic response synergetically contributes to the development of endometrial lesions and relative clinical manifestations. Similar to PE, Ng et al. proposed that ischemia-reperfusion has occurred at nearby ectopic lesions in patients who experienced periodic retrograde menstruation, leading to periodic abdominal pain (Ng et al., 2020). This was attributed to the escalated generation of ROS, and activation of robust inflammatory response due to the over-production of pro-inflammatory mediators and leukocytes (Ng et al., 2020). Moreover, EESCs undergoing ferroptosis were found to stimulate angiogenesis of surrounding tissues by paracrine secretion of vascular endothelial growth factor and IL-8 which are two key angiogenic factors (Li et al., 2022). This might contribute to the proliferation of the begin cells and the progression of EMs. Furthermore, increased iron availability of follicular fluid in granulosa cells (GCs) is possibly linked to the failure of oocyte maturation, reduction of ovarian reserve, and abrogation of blastocysts development, further resulting in female infertility (Chen et al., 2021a; Li et al., 2021a; Ni et al., 2022).

## 5 The roles of ferroptosis in PCOS

PCOS is one of the most frequent and prevalent gynecological conditions among women, characterized by hyperandrogenism, insulin resistance and polycystic ovarian morphology (Hoeger et al., 2021). One of the major features of PCOS is the follicular excess attributed to failure of normal follicular degeneration termed as follicular atresia (Dewailly et al., 2014). Over the past few years, more evidence has suggested that ferroptosis might be involved in the normal follicular atresia and provide a novel therapeutic strategy for PCOS management.

### 5.1 Pre-existing ferroptosis in normal follicular atresia

Traditionally, it was believed that follicular atresia was dominantly triggered by apoptosis of GCs with an orchestrated balance of pro-survival and pro-apoptotic factors, whereas recent studies revealed that ferroptosis also involves in this process (Kaipia and Hsueh, 1997; Sugiyama et al., 2015; Robker et al., 2018). Firstly, Zhang et al. reported a reduced level of TF in the atretic follicles, indicating the iron content in GCs was significantly upregulated compared to healthy follicles (Zhang et al., 2018). Secondly, activation of steroidogenesis in atretic follicles is potential to induce ferroptosis. Ovarian steroidogenesis is known to be activated in GCs to initiate the atresia, causing an increase in androgen production and a decrease in estradiol (Zhang et al., 2018). In an integrated proteomic and metabolomic analysis of chicken ovary, researchers demonstrated that lipoprotein lipase is upregulated in sexually matured chicken and extensively enriched in the glycerolipid metabolism pathway, implying an internal connection between steroidogenesis and lipid accumulation of GCs during the maturation of ovarian follicles (Cui et al., 2022). Additionally, similar to GCs, Weigand et al. found that active steroidogenesis is the potential cause for lipid peroxidation in adrenal cortex cells (Weigand et al., 2020). Although steroidogenesis has suggested its role in inducing susceptibility to ferroptosis in atretic follicles, the exact molecular mechanism remains poorly understood, which warrants further investigation. Thirdly, the depletion of GSH was detected in the early atretic follicles. Rabah et al. found GSTA1 and 2 which conjugate with GSH was upregulated only in GCs from healthy follicles, enhancing the detoxification effect of GSH (Rabahi et al., 1999). Terenina et al. also elucidated that more active transcriptions of glutathione S-transferase A1 (GSTA1) and glutathione S-transferase A2 (GSTA2) were present in early healthy follicles, further reducing the accumulation of ROS (Terenina et al., 2017). In this regard, GC in early atretic follicles is more vulnerable to ferroptosis due to less active GSH compared to GC in healthy follicles.

### 5.2 Ferroptosis and PCOS therapy

**FIGURE 4:**
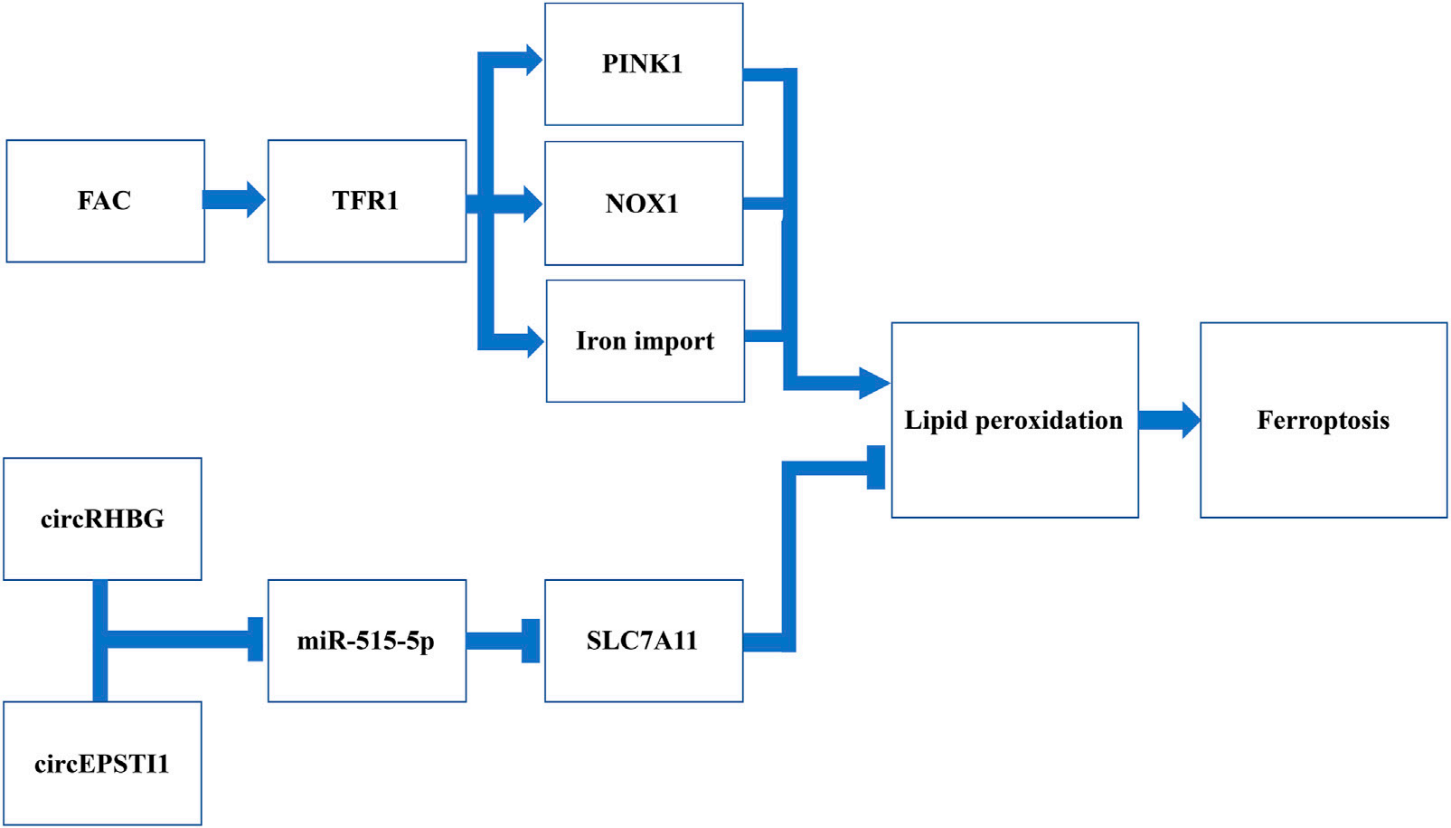
Schematic representation of potential targets triggering ferroptosis for PCOS management.Induction of FAC is suggested to promote iron import by increasing the expression of TFR1 in DHEA-injected mice. In addition, the induction of FAC has been demonstrated to further activate the NOX1 and PINK1, two factors inducing ROS and ultimately lipid hydroperoxides. CircRHBG and circEPSTI1 are two circRNA that can inhibit miR-515-5p and further de-suppress the expression of SLC7A11, one of the system Xc^−^ subunits. Therefore, silencing these two circRNA can reduce the expression of system Xc^−^, thus leading to antioxidants depletion. Collectively, both induction of FAC and silencing circRHBG as well as circEPSTI1 have been revealed to promote lipid peroxidation and trigger ferroptosis.

Current treatments for PCOS exclusively focus on the management of symptoms, whereas investigations of its long-term treatments are still limited. Due to the vital role of ferroptosis implicated in follicular atresia, it has become the spotlight for developing novel therapeutic modalities for the long-term management of PCOS (Figure 4).

#### 5.2.1 Inducing ferroptosis *via* iron metabolism

As iron metabolism is key to the regulation of ferroptosis, recently it has been receiving an increasing attention in ferroptosis-mediated PCOS therapy. Ferric ammonium citrate (FAC), a TFR1 inducer which can promote iron import, has been shown to ameliorate the formation of polycystic ovaries in DHEA-injected mice (Zhang et al., 2021a). In addition, an increase of iron uptake can further activate NADPH oxidase 1 (NOX1), another factor that can increase the susceptibility to ferroptosis by inducing ROS (Dixon et al., 2012). TFR1 can activate the PTEN-induced kinase 1 (PINK1) signaling, which causes mitophagy and accumulation of lipid peroxides, resulting in depletion of GPX4 and finally ferroptosis (Zhang et al., 2021a). The experiment conducted by Zhang et al. have found that, following the additional use of FAC in DHEA-injected mice, the restored ovarian function along with the increased number of embryos implanted was reported (Zhang et al., 2021a). This suggests that inducing ferroptosis *via* upregulating TFR1 expression can be a promising strategy for managing ovulation in PCOS patients.

#### 5.2.2 Inducing ferroptosis *via* targeting circRNA

CircRNA is a type of non-coding RNA and is capable to regulate genetic expression *via* binding to microRNA (miRNA) (Ebbesen et al., 2016; Liu et al., 2020). Previous studies have found that circRNA-miRNA interaction significantly alters cell inflammation, infection and OS (Ebbesen et al., 2016; Liu et al., 2020). Therefore, targeting circRNA might change the lipid oxidative status and antioxidative system, which in turn affect ferroptosis.

Recently, with the continuous investigation of the interaction between circRNA and miRNA in PCOS patients, Zhang et al. reported that overexpression of circRHBG (circRNA originates from RHBG) could inhibit ferroptosis, leading to ovulation failure (Zhang et al., 2021b). It has been suggested that circRHBG can interact with miR-515-5p, the tumor suppressor that can downregulate SLC7A11 expression (Zhang et al., 2021b). The upregulated circRHBG competes with SLC7A11 to bind to miR-515-5p, leading to overexpression of SLC7A11 (Zhang et al., 2021b). In addition, the knockdown of circRHBG was also found to reduce the GSH/GSSG ratio, which promotes ROS accumulation (Zhang et al., 2021b). Likewise, another research focused on cervical cancer has demonstrated that silencing circEPSTI1 which also targets miR-515-5p. This leads to suppressed tumor growth in mouse xenograft models with an increased lipid peroxides on the tumor cell membrane (Wu et al., 2021). They also observed a reduced GSH/GSSG ratio and the expression of GPX4, further indicating the susceptibility to ferroptosis in cervical cancer cells (Wu et al., 2021). CircEPSTI1 might be effective in PCOS as it targets the same miRNA, namely, circRHBG, which requires further investigations. In recent years, genetic therapy *via* RNA interference has shown promising prospects in various fields, however, studies focused on PCOS management are still insufficient. These research findings indicated that circRHBG and circEPSTI1 might be a potential treatment target and candidate of prognosis markers in PCOS.

## 6 Conclusion and future directions

Since the discovery of ferroptosis in 2012, it has been demonstrated to be associated with several human diseases pathologically and therapeutically, especially obstetrical and gynecological diseases. Impaired iron homeostasis, depletion of intracellular antioxidants and overproduction of lipid hydroperoxides are three major characteristics of ferroptosis.

Ferroptosis is suggested to be highly involved in the pathogenesis of an early-onset type of PE with excessive accumulation of iron and lipid peroxides (Siddiqui et al., 2013; Aouache et al., 2018; Liu et al., 2019; Yang et al., 2020; Chen et al., 2022). Additionally, the inhibition of anti-ferroptosis factors can trigger the pathogenesis of PE *in vitro* (Beharier et al., 2020; Meihe et al., 2021). Ferroptosis has also been demonstrated to contribute to inflammation in PE patients. Suboptimal spiral artery remodeling, one key feature of PE pathology is also affected by ferroptosis *via* suppression of trophoblast differentiation (Sangkhae et al., 2020). Ferroptosis is closely associated with inflammatory response and hemodynamic abnormalities, the interactions among these three elements synergistically contribute to the systemic vascular destruction and further clinical symptoms of PE.

Ferroptosis has been implicated in a crucial role in the pathogenesis of EMs. Ferroptosis resistance enables endometrial cells to grow and survive with the presence of excessive iron and OS microenvironment (Ng et al., 2020). In contrast, at nearby ectopic lesions, the occurrence of ferroptosis cooperated with inflammation and angiogenesis contributes to the formation of subsequent lesions (Ng et al., 2020; Li et al., 2022).

It has been demonstrated that the initiation of ovarian follicular atresia is largely dependent on ferroptosis, the abrogation of which leads to failure of ovulation (Rabahi et al., 1999; Terenina et al., 2017; Zhang et al., 2018; Cui et al., 2022). Likewise, induction of ferroptosis might provide a novel therapeutic strategy for managing ovulation in PCOS patients. *In vivo* studies have suggested that either induction of TFR1 or circRHBG holds promising prospects in this field (Zhang et al., 2021a; Zhang et al., 2021b; Wu et al., 2021).

Collectively, ferroptosis can be considered to have dual characters. It exerts detrimental effects on female reproductive systems as it contributes to a series of obstetrical and gynecological diseases. Meanwhile, due to its potential therapeutic effects, it can benefit patients who suffer from reproductive diseases currently with disappointing management.

Till now, with the deepening investigation of ferroptosis in various female reproductive diseases, the involvement of ferroptosis in PE, PCOS and EMs becomes increasingly apparent, and targeting ferroptosis of these diseases in the clinical implementation has a theoretical feasibility. However, several unanswered questions hinder the ferroptosis-targeting therapy from reaching final applications. Given the dual role of ferroptosis presenting in the distinct female reproductive diseases, the precise and accurate interpretation of pathophysiological roles of ferroptosis at the cellular, tissue and systemic levels is critically required to select either ferroptosis-induction or ferroptosis-inhibition regimen for those certain diseases. Moreover, the systemic understanding of the interaction between ferroptosis and its potentially associated pathological process, such as hemodynamics and inflammation, is of great importance, as targeting these pathways simultaneously might improve therapeutical effects. Notably, ferroptosis is an intricate iron-dependent process that could occur in both physiological and pathological conditions in multiple organs. Therefore, the specific targeting of ferroptosis regulators to particular cells and the precise delivery to certain organs must be guaranteed, which requires further pharmaceutical investigations. Additionally, emergent evidence has shown that ferroptosis can be considered with iron-dependent regulatory necrosis, tightly associated with several cell death pathways, including autophagy, pyroptosis, necrosis, and apoptosis (Yee et al., 2020; Ma et al., 2022), but the exact molecular interaction has not been fully elucidated at present. Therefore, it is critical to unravel their interplays to facilitate the designing of novel therapeutic measures with the integrated regulation of several cell death pathways. Taken together, the application of ferroptosis-targeting therapy in the clinical fields is both challenging and promising. Resolving these unanswered questions might help gain deeper insight not only into the clinical implementation of ferroptosis, but also into the various areas of biology to which it is tightly linked.

## Glossary

AA: arachidonic acid
ACSL4: acyl-CoA synthetase long chain family member 4
ALOXs: arachidonate lipoxygenases
DAMPs: damage-associated molecular patterns
DMT1: divalent metal transporter
EESCs: ectopic endometrial stromal cells
EMs: endometriosis
EVTs: Extravillous trophoblast cells
FAC: Ferric ammonium citrate
Fe2+: Ferrous iron
Fe3+: Ferric iron
FPN: Ferroportin
GCs: Granulosa cells
GPX4: Glutathione peroxidase 4
GSH: Glutathione
GSSG: Glutathione disulfide
GSTA1: Glutathione S-transferase A1
GSTA2: Glutathione S-transferase A2
HIF-2α: Hypoxia-inducible factor-2α
4-HNE: 4-hydroxynonenal.
IL: Interleukin
IREB2: Iron response element binding protein 2
LOX: Lipoxygenase
LPCAT3: Lysophosphatidylcholine acyltransferase 3
MDA: Malondialdehyde
MDR: Multiple drug-resistant
miRNA: microRNA
NCOA4: Nuclear receptor coactivator 4
NOX1: NADPH oxidase 1
OS: Oxidative stress
PARPi: Poly (ADP-ribose) polymerases inhibitor
PCBP: Poly rC binding protein 2
PCOS: Polycystic ovarian syndrome
PE: Preeclampsia
PHT: Primary human trophoblast
PINK1: PTEN-induced kinase 1
PLs: Phospholipids
POR: P450 oxidoreductase
PTGS: Prostaglandin-endoperoxide synthase
PUFA: Polyunsaturated fatty acid
PUFA-PL-OH: PUFA phospholipid alcohols
PUFA-PL-OOH: PUFA phospholipid hydroperoxides
ROS: Reactive oxygen species
Sat1: Spermidine/spermine N1-acetyltransferase 1
STEAP3: Six-Transmembrane Epithelial Antigen of Prostate 3
1; System Xc-: Sodium-independent, anion amino acid transport system
T-A: Total antioxidant capacity
TF: Transferrin
TFR1: Transferrin receptor 1.
